# Readiness on dental trauma in federally qualified health centers. Why it matters?

**DOI:** 10.21142/2523-2754-1303-2025-247

**Published:** 2025-08-31

**Authors:** Yessenia Valverde Ingersoll

**Affiliations:** 1 DDS, PhD. La Maestra Community Health Centers 4171. San Diego, CA., United States of America La Maestra Community Health Centers San Diego CA United States of America

Dental Trauma refers to injuries to the lips, teeth, gums and supporting bone due to accidents such as falls, injuries, or assaults. ^1^ Readiness for treating dental trauma is of upmost importance in Federal Qualified Health Centers (FQHCs) which serve medically underserved populations, often including children, low-income individuals, and those without regular access to dental care. ^2^

Although dental trauma may not always be the top priority in primary care settings, its consequences can be severe and long-lasting, particularly when treatment is delayed. ^(^^3^ For the vulnerable populations, FQHCs often act as the first point of contact for urgent dental needs. ^(^^4^ Early intervention is critical to preventing complications that may include infection, pain, and even tooth loss. ^3^

It is essential then that dentists in these settings are trained and equipped to respond promptly to dental emergencies and urgent needs. Specially, when it has been stablished association between low socio-economic status and dental injury. ^5^ Correlating to the type of population served in FQHCs. This readiness can help prevent minor injuries from escalating into serious public health issues such as teeth loss. ^3^

While dental trauma may not always be classified as a full emergency, it should be recognized as an urgent care priority that warrants timely attention. Typically, dental trauma appointments are unplanned and require immediate attention. In many cases, the dentist may have only 30 minutes or less (in case of tooth avulsion) to promptly assess the situation, provide an accurate diagnosis, and intervene to prevent complications such as tooth loss. This limited window of time makes preparation and readiness critical. ^6^^,^^7^

By addressing dental trauma proactively-through training, protocols, and resource allocation-FQHCs can strengthen their role as providers of equitable and comprehensive care, ultimately improving oral health outcomes for some of the nation’s most vulnerable and underserved populations.

Many FQHCs employ general dentists, hygienists, and sometimes pediatrics dentists. However, ensuring that the clinical staff are trained in dental trauma management (e.g., avulsion replantation protocols, splitting techniques) enhances care quality. Thus, having standard operating procedures (SOPs), emergency kits, and referral networks in place is key to readiness. ^8^

What is greatly needed is the training and continuing education on dental trauma and stocking on emergency kits and materials (e.g., splints, calcium hydroxide, storage media like Hank’s Balanced Salt Solution), as well as developing referral system with nearby specialist such Endodontists or Oral Surgeons, and Implementing documentation with follow-up protocols by following IADT guidelines. ^7^

In summary, readiness to manage dental trauma in FQHCs is not just a clinical issue-it’s a health equity issue. By ensuring proper training, protocols, and infrastructure, FQHCs can protect vulnerable populations from preventable dental complications, reduce emergency department system burdens, and improve community trust and outcomes in oral healthcare.
